# Paper- vs. computer-based assessment in high-stakes testing: a common-person investigation of mode comparability

**DOI:** 10.3389/fpsyg.2026.1867992

**Published:** 2026-09-08

**Authors:** Kübra Atalay Kabasakal, Tuba Gündüz

**Affiliations:** 1 Hacettepe University, Faculty of Education, Ankara, Türkiye; 2 Muğla Sıtkı Koçman University, Faculty of Education, Muğla, Türkiye

**Keywords:** Common-person design, construct equivalence, high-stakes assessment, mode comparability, Rasch model, score comparability, separate and concurrent calibration, test equating

## Abstract

This study examined the mode comparability of paper-based (YDS) and computer-based (e-YDS) forms of a high-stakes foreign language examination administered in Türkiye. Rather than treating the analysis as a routine test equating, we framed it as a test of whether a change in administration mode leaves the measured construct invariant and the resulting scores comparable. Using a common-person design and the Rasch model, six form pairs were placed on a common scale through both separate calibration with mean/sigma transformation and concurrent calibration. Model–data fit and dimensionality (via tetrachoric inter-item correlations and parallel analysis) were evaluated for every form, and the linking was examined with and without the exclusion of inconsistent examinees as a sensitivity analysis. Scaling constants, the linking RMSE, and the paired mean difference (Δ) were computed, and cross-form test information functions were compared. Across all pairs the data were essentially unidimensional and fit the Rasch model adequately, with a comparably dominant single dimension for paper-based and computer-based forms. Although the computer-based forms did not display a stronger secondary dimension than the paper-based forms, the design—with different items and administration dates—cannot isolate a pure mode effect. Linking constants and the estimated mode difference were nearly identical with and without examinee exclusion (mode differences ≤ ~0.17 logits), and the separate and concurrent methods agreed almost perfectly at the item-parameter level (*r* = 1.00). Computer-based forms yielded slightly higher ability estimates, but the common-examinee distributions remained stable. Taken together, the results provide evidence of practical mode comparability under the present operational conditions, while we are explicit about the confounds and assumptions that bound this conclusion.

## Introduction

In large-scale assessments, multiple forms of the same test are typically developed to protect test security and fairness (Wu et al., 2025; American Educational Research Association, 2014). Two conceptually distinct problems must be separated at the outset. The first is alternate-form equating: when forms built from different item pools differ slightly in difficulty, a statistical transformation places their scores on a common scale so that examinees are neither advantaged nor disadvantaged by the particular form they receive (Holland and Dorans, 2006; Dorans, 2004; Zhu, 1998). Equating, however, presupposes that the forms measure the same latent construct with the same structural properties. The second problem—central to the present study—is mode comparability: whether delivering a test on paper rather than on a computer leaves the measured construct and the comparability of scores intact. If the change of mode alters item difficulty or introduces construct-irrelevant variance (for example, through interface friction, on-screen navigation, or screen fatigue), this is a question of construct validity and measurement invariance that equating cannot simply absorb. We therefore treat equating as a tool for placing scores on a common metric, while the substantive question we investigate is whether the paper-based and computer-based forms are mode-comparable. Because the YDS is a high-stakes examination—its scores determine eligibility for graduate study, academic promotion, and public-service language requirements—even small, mode-related distortions can be consequential, which is precisely why mode comparability must be established empirically.

Large-scale examinations are increasingly administered electronically, complementing or replacing paper-based delivery. A clarification is important here, because the literature spans two fundamentally different paradigms. Following Ma et al. (2025), one must distinguish digitized (linear) assessments—formats that mirror a paper test on screen with one-to-one item correspondence and an unchanged blueprint—from advanced, interactive digital assessments that exploit technology to measure constructs in ways impossible on paper. The YDS and e-YDS comparison examined here falls strictly within the digitized-linear category: the test specifications, blueprint, length, and time limit are maintained across administrations, but the individual items differ form to form and the two modes were administered on different dates; consequently, observed differences cannot be attributed to administration mode alone, and our claims are confined to this digitized-linear paradigm. While early work established the feasibility of computer-based large-scale testing (Breiter et al., 2013; Koomen and Zoanetti, 2016), computer-based delivery has since become institutionalized across national and professional testing systems (Karkenova, 2025; Pai, 2025; Tuna Pusa and Dinçer, 2025; Perry et al., 2022). Digitized delivery offers efficiency, faster scoring, and logistical flexibility, but it also raises the central question of this study: can scores from paper-based and computer-based forms be used interchangeably for high-stakes decisions?

By situating a psychometrically rigorous mode-comparability study within a broader validity and replicability discourse, this paper makes explicit what such a design can legitimately support and, equally importantly, what it cannot. In doing so, it contributes to methodological reflection in psychometrics by showing how early design choices delimit—rather than guarantee—claims of equivalence, fairness, and interchangeability in high-stakes testing.

## Test equating and comparability of administration mode

Equating methods are generally categorized into Classical Test Theory (CTT)-based and Item Response Theory (IRT)-based approaches. IRT-based equating involves a multistep process: ensuring model–data fit, selecting an appropriate design, estimating item and ability parameters, and aligning them onto a common scale. Among IRT-based designs, single-group, random-group, and common-item nonequivalent-group designs are the most common (Cook and Eignor, 1991; Kolen and Brennan, 2004). When no anchor items are shared across forms, common-examinee (person) designs are used. In this design, the same group of test takers completes two forms, so stable between-person differences in ability are controlled; ability that changes between administrations, however, is not (Cook and Eignor, 1991; Yu and Popp, 2005).

Throughout this paper we use the term equivalence in a deliberately specified sense, because it is otherwise easily conflated. Three nested levels are commonly distinguished. Construct equivalence concerns whether the same latent dimension and internal structure underlie performance in both modes. Measurement invariance concerns whether item parameters—here, Rasch item difficulties—remain stable across modes. Score equivalence concerns whether the resulting scores can be treated as interchangeable for decision-making. Measurement invariance presupposes construct equivalence, and score equivalence is meaningful only once the former two are reasonably supported. The present study provides evidence primarily at the levels of construct equivalence and overall score comparability—through model fit, dimensionality, and the stability of linking constants. Because the paper- and computer-based forms share no common items, item-level invariance (differential item functioning) cannot be assessed, so we do not claim strict measurement invariance; the operational YDS is in any case reported as a number-correct scaled score rather than on the logit metric.

In test equating, score comparability is typically achieved through either common-item or common-person equating (Holland and Dorans, 2006; van der Linden and Hambleton, 1997). Relative to common-item equating, the common-person design has been applied less frequently in operational high-stakes settings, largely because it requires administering two full-length forms to the same examinees within a short window (Yu and Popp, 2005; Boone and Staver, 2020; Liu et al., 2025); where it has been used to study mode effects, samples of common examinees have often been modest. The present study contributes a large set of common examinees (745 to 2,464 per pair) drawn from an operational high-stakes program, which strengthens the stability of the linking relative to earlier, smaller-scale applications.

As assessments transition from paper to computer, ensuring comparability across modes has become a critical issue in test validation. Mode effects can stem from user-interface differences, item presentation, examinee familiarity with digital tools, or environmental conditions (Bennett, 2003). If not properly addressed, such differences may threaten construct equivalence, advantaging or disadvantaging particular groups.

Early research on mode comparability focused on low-stakes international assessments such as PISA and TIMSS, where comparability was generally supported under carefully controlled conditions (Fishbein et al., 2018; Jerrim et al., 2018). More recent work underscores the need for comparable research in high-stakes contexts, where small performance differences can carry significant consequences (Perry et al., 2022; Zhai and Pellegrino, 2023). Empirical findings by Kingston (2009) and Wang et al. (2007) show that even minor interface differences can affect item functioning and engagement. Validating equivalence across formats is therefore both a psychometric necessity and an ethical imperative. While mode differences may diminish as technology and examinees adapt, this assumption must be tested empirically rather than presumed (von Davier et al., 2020; Wiberg and Laukaityte, 2025).

## YDS and e-YDS in Türkiye

This study examines the comparability of paper-based and computer-based formats of a high-stakes, large-scale assessment: the Foreign Language Examination (YDS) and the electronic Foreign Language Examination (e-YDS), both administered by the Republic of Türkiye Center for Assessment, Selection, and Placement (ÖSYM). The acronym YDS (Yabanci Dil Sinavi) reflects the initials of the examination's full Turkish title.

The YDS is used for several purposes, including qualification as a foreign-language teacher candidate, admission to graduate programs, eligibility for associate professorship, and language compensation in public service. Scores are valid for 5 years, and candidates may retake the test (ÖSYM, 2025a). This study focuses on the English version. Since 2013 the YDS has been administered twice annually on paper; since 2014 the computer-based e-YDS has been offered up to seven times a year, exclusively in English. The e-YDS was introduced to align with the increasing digitalization of large-scale assessment in Türkiye and to reduce the cost and logistical burden of administration, while preserving the same item blueprint and test specifications as the paper-based YDS.

Although both formats contain 80 items and impose a 180-min limit, they differ in important ways. The e-YDS provides faster results and greater scheduling flexibility, whereas the paper-based YDS offers broader geographic accessibility: more than 200,000 candidates can sit the YDS nationwide, while the e-YDS is limited to roughly 5,000 candidates across four designated provinces due to infrastructure constraints (ÖSYM, 2025a,b). The e-YDS also reduces clerical errors by preventing multiple selections per item and eliminating answer-sheet transfer problems common to the paper format.

Equating was conducted using e-YDS forms administered both before and after each paper-based YDS in 2024. The March YDS (YDS1) was linked with the February (e-YDS2) and April (e-YDS4) e-YDS sessions, and the November YDS (YDS2) with the October (e-YDS11) and December (e-YDS12) sessions. The administration dates were e-YDS2 (11 February 2024), YDS1 (24 March 2024), e-YDS4 (27 April 2024), e-YDS11 (19 October 2024), YDS2 (3 November 2024), and e-YDS12 (21 December 2024); the intervals between linked administrations ranged from 15 to 76 days (Table 1). Short, sequential timing was chosen to limit—though it cannot eliminate—change in examinee ability between sittings.

**Table 1:** Sample Sizes, Common Examinees, and Administration Intervals for Each Form Pair (common-examinee counts are reported both before and after outlier exclusion).

Pair	Form X	Form Y	Form X	Form Y	Common	Common after outlier removal	Interval (days)
RQ1a	YDS1	e-YDS2	4,387	3,572	1,892	1,802	42
RQ1b	YDS1	e-YDS4	4,699	3,605	2,207	2,104	34
RQ2a	YDS2	e-YDS11	4,817	3,565	2,330	2,214	15
RQ2b	YDS2	e-YDS12	4,957	3,840	2,464	2,358	48
RQ3	e-YDS2	e-YDS4	3,225	3,198	745	702	76
RQ4	e-YDS11	e-YDS12	3,209	3,343	922	885	63

Because the forms share no common items, a common-person design was employed, in which the same individuals took both a paper-based and an electronic form. The design is thus a common-person linking design in which examinees, not items, serve as anchors. Within this design we used two complementary routes to a common scale and compared them: separate calibration with transformation, and concurrent calibration. In separate calibration each form is calibrated independently and the first form is treated as the reference scale, to which the second form is aligned using mean/sigma constants derived from the common examinees. In concurrent calibration both forms are calibrated in a single run, placing all parameters on one metric without a separate transformation step. Following Hanson and Béguin (2002), the two methods are compared as alternative means of recovering the same common scale.

## Research questions

Within an IRT framework and a common-person design, the study addressed the following questions. (1) To what extent are the paper-based YDS1 and the electronic e-YDS2 and e-YDS4 forms mode-comparable? (2) To what extent are YDS2 and the electronic e-YDS11 and e-YDS12 forms mode-comparable? (3) Are the electronic forms e-YDS2 and e-YDS4 comparable with each other? (4) Are e-YDS11 and e-YDS12 comparable with each other? Linking paper-based forms with computer-based forms administered immediately before and after them records the direction of administration, while linking electronic forms with one another helps to separate mode from timing and content; order effects were not formally tested, and administration direction is treated as a descriptive feature rather than a controlled factor. Owing to the long interval between the two paper-based forms, YDS1 and YDS2 were not linked.

Given the goal of aligning forms on the basis of a shared sample of examinees, the Rasch (1PL) model was selected. Three considerations motivated this choice: its parameter-separation and specific-objectivity properties suit common-person linking; its equal-interval logit metric supports mean/sigma scale alignment; and a parsimonious model is preferable when the linking sample, rather than item discrimination, is the focus. Because ÖSYM does not publish an operational IRT scoring model for the YDS—scores are reported on a number-correct basis—the Rasch analysis here is a research re-calibration whose adequacy must be demonstrated empirically rather than assumed. Accordingly, model fit and the dimensionality of the item responses (assessed with tetrachoric inter-item correlations and parallel analysis) are reported in the Results, and the unidimensionality assumption is examined directly against the possibility that interface differences introduce a secondary dimension.

## The Rasch model

The Rasch model is a probabilistic IRT model widely used in educational measurement for its simplicity and theoretical properties. It places person and item parameters on a common equal-interval logit scale and, when the model fits, yields estimates that are invariant across samples and forms—the property of specific objectivity (Wei et al., 2014) that underlies equating across modes. The probability of a correct response is Equation 1


$$\begin{array}{c} P\left(\;X=1|\;B_{n},\;D_{i\;}\right)=\;\frac{e^{\left(B_{n}\;-\;D_{i\;}\right)}}{1+\;e^{\left(B_{n\;}-\;D_{i}\right)}} \end{array}$$ (1)

where *B*_*n*_ is the ability of person *n* and *D*_*i*_ is the difficulty of item *i*. This equation indicates that the probability of a correct response increases as a person's ability surpasses item difficulty. The log-odds form of this equation is given by Equation 2:


$$\begin{array}{c} \ln\;\left(\frac{P_{ni}}{1-\;P_{ni}}\right)\;=\;B_{n\;}-\;D_{i} \end{array}$$ (2)

As a logistic regression model, the Rasch framework allows both item and person parameters to be aligned on a single underlying scale, typically ranging between −4 and +4 logits.

## Common-person equating

In common-person equating the same individuals complete two forms and serve as natural anchors for aligning the scales (Boone and Staver, 2020). Person abilities were first estimated separately for each form using the Rasch model. To assess the stability of the linking, a regression was fitted between each examinee's ability estimates on the two forms, and cases with standardized residuals greater than 2 in absolute value were flagged (Linacre, 2011b). We emphasize that these cases were not deleted to manufacture agreement; rather, the linking is reported both with and without them as a sensitivity analysis, and the primary results are based on the full common-person sample.

To place parameters on a common scale, both separate calibration with transformation and concurrent calibration were used. In separate calibration the two forms are calibrated independently and a linear transformation aligns the ability scales Equation 3,


$$\begin{array}{c} \theta^{*}=\text{A}\cdot \theta \;+\text{B}, \end{array}$$ (3)

where A and B are the scaling coefficients (Kolen and Brennan, 2004, 2014). Following a mean/sigma approach—but using the means and standard deviations of the common examinees' abilities rather than common items—the constants are Equation 4


$$\begin{array}{c} \text{A}=\;\sigma \left(\text{X}\right)/\sigma \left(\text{Y}\right),\;\text{B}=\;\mu \left(\text{X}\right)\;-\text{A}\cdot \mu \left(\text{Y}\right), \end{array}$$ (4)

where X denotes the reference (first) form and Y the form being transformed. The regression described above was used only to flag inconsistent examinees, not as the linking method. In contrast, concurrent calibration estimates parameters for both forms in a single analysis, eliminating the transformation step (Hanson and Béguin, 1999, 2002; Boone and Staver, 2020; Linacre, 2011b). In this joint calibration the responses of all examinees to all 160 items (the 80 items of each form) were analyzed together in one estimation, so that every item difficulty and every person ability—including freely estimated abilities for the common examinees, who responded to both forms—were placed on a single common metric without a separate transformation. Because concurrent calibration already yields a common metric, we do not compare “new” against “original” ability estimates—a comparison that would chiefly reflect rescaling and estimation noise—but instead evaluate the agreement between the two methods at the item-parameter level (see Results).

## Method

## Participants and data sources

Data were drawn from two paper-based administrations (YDS1, YDS2) and four electronic administrations (e-YDS2, e-YDS4, e-YDS11, e-YDS12) of the 80-item English YDS in 2024. Because far more candidates take the paper-based YDS than the e-YDS, random samples of YDS examinees were drawn to match each e-YDS administration. This matching was adopted to give the two marginal groups comparable size and calibration precision and to prevent the very large paper-based sample from dominating the joint estimation; because the scaling constants are derived from the common examinees rather than from the marginal groups, the matching has limited bearing on A and B. We could not re-calibrate on the full national YDS population, which was not available to us, and we note this as a limitation. Table 1 summarizes the sample sizes, the number of common examinees, and the administration intervals for each pair.

**Figure 1:**
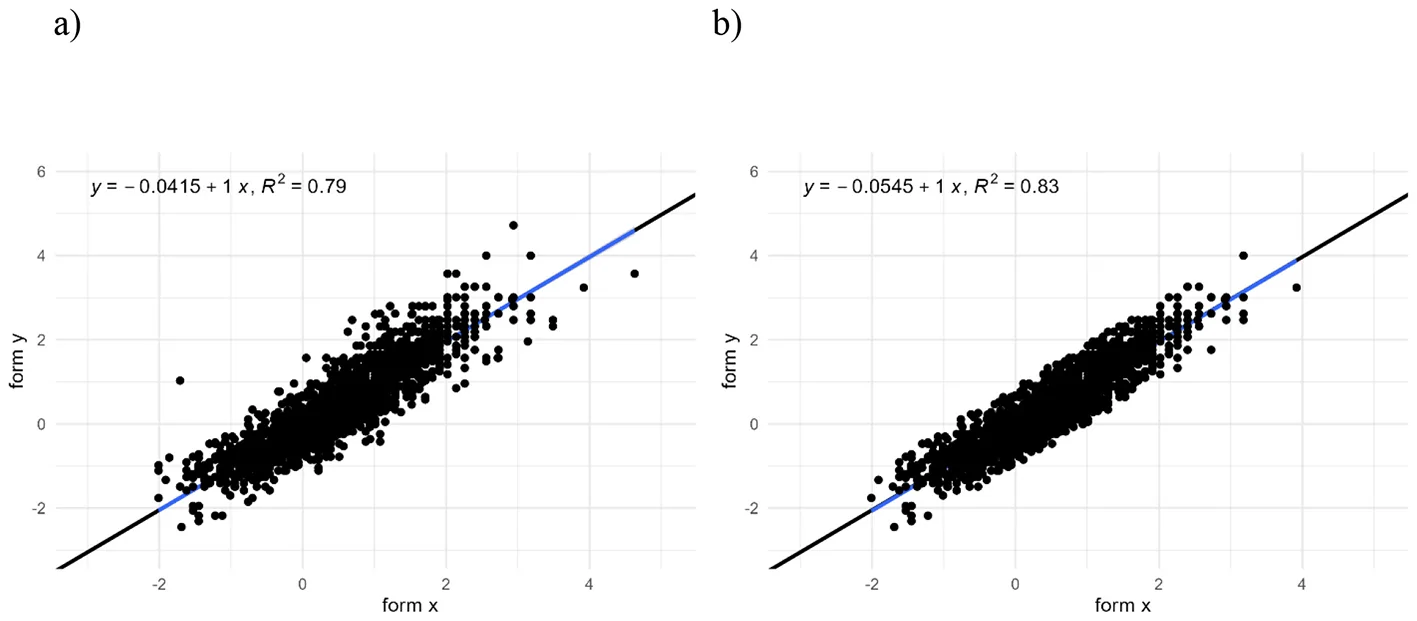
Common-person ability estimates for YDS1 and e-YDS2, before and after exclusion of inconsistent cases.

Item and ability parameters for each form were estimated with the Rasch model in Winsteps (Linacre, 2011a). Across pairs, the flagged (standardized residual>|2|) examinees represented only 4.0–5.8% of common examinees (e.g., 103 of 2,207 for YDS1–e-YDS4). Figure 1 illustrates the common-person scatter before and after exclusion for the YDS1–e-YDS2 pair.

Table 2 reports descriptive statistics for ability estimates on each form and for the common-person subsets, after exclusion of flagged cases.

**Table 2:** Descriptive statistics of ability estimates by form and common-person subset.

Pair		Form	*n*	Mean	sd	Min	Max
RQ1a	YDS1-e-YDS2	YDS1	4297	0.14	1.11	−3.47	5.85
		e-YDS2	3482	0.52	1.24	−4.67	5.94
		common_YDS1	1802	0.42	0.88	−2.01	3.92
		common_e-YDS2	1802	0.37	0.97	−2.45	4.00
RQ1b	YDS1-e-YDS4	YDS1	4596	0.18	1.11	−4.61	4.63
		e-YDS4	3502	0.52	1.10	−2.61	5.92
		common_YDS1	2104	0.42	0.89	−2.55	3.18
		common_e-YDS4	2104	0.53	0.96	−1.85	3.98
RQ3	e-YDS2-e-YDS4	e-YDS2	3182	0.55	1.28	−4.67	5.94
		e-YDS4	3155	0.52	1.13	−2.60	5.92
		common_e-YDS2	702	0.55	1.01	−1.95	4.72
		common_e-YDS4	702	0.70	0.92	−2.06	3.98
RQ2a	YDS2-e-YDS11	YDS2	4701	−0.03	1.16	−3.49	5.85
		e-YDS11	3449	0.43	1.06	−2.83	4.69
		common_YDS2	2214	0.30	1.00	−2.02	3.92
		common_e-YDS11	2214	0.38	0.91	−1.87	3.24
RQ2b	YDS2-e-YDS12	YDS2	4851	−0.08	1.11	−3.50	4.63
		e-YDS12	3734	0.35	1.09	−2.41	5.83
		common_YDS2	2358	0.17	0.94	−2.56	3.91
		common_e-YDS12	2358	0.33	0.91	−1.81	3.90
RQ4	e-YDS11-e-YDS12	e-YDS11	3172	0.45	1.08	−3.04	4.69
		e-YDS12	3306	0.36	1.12	−2.41	5.83
		common_e-YDS11	885	0.50	0.89	−1.59	3.24
		common_e-YDS12	885	0.51	0.90	−1.62	3.90

Across pairs, mean ability estimates were somewhat higher for the computer-based forms than for the paper-based forms. This pattern is consistent with a selection difference rather than mode alone: the paper-based YDS is administered nationwide to a broad population, whereas the e-YDS is offered in a limited number of metropolitan centers and may attract a somewhat more prepared or computer-confident population.

The common-person design controls for stable between-person ability differences by using the same examinees as anchors. This control is not absolute, however. Because the linked forms were administered days to weeks apart (15–76 days; Table 1), examinees may have studied, gained test familiarity, or experienced fatigue or motivational shifts between sittings, so the latent trait is not perfectly invariant over time. Such temporal change adds unmodeled variance to the person anchors and can attenuate the linking relationship; we therefore interpret the linking constants as approximations obtained under a near-invariance assumption. Reassuringly, the common-person subsets exhibited more homogeneous ability distributions than the full groups, with narrower standard deviations and means clustered more closely together (Table 2), supporting their use as a basis for scale alignment. A few estimates beyond ±4 logits reflect examinees at ceiling or floor. Administration within a controlled environment, with verified examinee identity and standardized conditions, further limits extraneous sources of variance.

## Equating procedure and linking-discrepancy metrics

The aim of the analysis is to determine whether scores from forms intended to measure the same construct can be treated as comparable across modes. Several indicators were computed. First, scaling constants A and B from separate calibration were obtained. All metrics were computed on the full common-examinee sample; the trimmed sample (the common persons remaining after exclusion of inconsistent cases) is used only for the sensitivity analysis (Table 3). Let θ_*X*_*j*__ and θ_*Y*_*j*__ denote the untransformed ability estimates of common examinee j from the two separate calibrations, and let $\text{RMSE }=\;\sqrt{\frac{\Sigma_{j}\;\left(\theta_{j}*\;-\theta_{Y_{j}}\right)}{N}}$ denote the form-Y estimate transformed to the form-X (reference) scale by mean/sigma. The linking RMSE is the root-mean-square discrepancy between the transformed and the original form-Y estimates and describes the magnitude of the change introduced by the transformation rather than accuracy against a known true value. The paired mean difference is Δ = mean(θ_*X*_*j*__−θ_*Y*_*j*__). Because A and B are estimated from these same common examinees, the transformed form-Y mean equals the form-X mean, so mean ($\text{RMSE }=\;\sqrt{\frac{\Sigma_{j}\;\left(\theta_{j}*\;-\theta_{Y_{j}}\right)}{N}}$ by construction—a TOST on this transformed difference would be uninformative. The mean of the transformed minus the original form-Y estimate is not zero; it equals Δ $\text{(mean(}\theta_{Y_{j}}*-\theta_{Y_{j}}))=\mu_{X}-\mu_{Y}=\Delta )$. We therefore report and test Δ itself. Δ is the systematic difference between the same examinees' estimates on the two forms and, equivalently, the location shift that the linking must apply (Δ ≈ B when A ≈ 1); it estimates the average form-plus-mode difference. Because the same persons take both forms and the A coefficients are close to unity (0.89–1.08), the two Rasch scales share essentially the same logit unit and differ mainly in origin, so Δ is interpreted as the mean logit shift between forms—a pre-linking systematic difference (the quantity the linking corrects) rather than a residual difference after linking (which is zero by construction). For concurrent calibration—which places all parameters on one metric in a single estimation and involves no (Equation 3) transformation—agreement with separate calibration was assessed at the item-parameter level rather than through a θ-to-θ comparison. The formulas are

**Table 3:** Sensitivity of linking to exclusion of inconsistent examinees.

Pair	% excluded	r full	r trimmed	Δ full (θ_X_-θ_Y_)	Δ trimmed (θ_X_-θ_Y_)
RQ1a	4.8	0.887	0.911	0.040	0.053
RQ1b	4.7	0.904	0.926	−0.114	−0.110
RQ2a	5.0	0.905	0.928	−0.087	−0.085
RQ2b	4.3	0.900	0.919	−0.173	−0.161
RQ3	5.8	0.895	0.920	−0.158	−0.153
RQ4	4.0	0.899	0.920	−0.020	−0.007


$$\text{RMSE }=\;\sqrt{\frac{\Sigma_{j}\;\left(\theta_{j}*\;-\theta_{Y_{j}}\right)}{N}}$$ (5)

$$\begin{array}{c} \Delta \;=\;\frac{\Sigma_{j}\;\left(\theta_{X_{j}}\;-\theta_{Y_{j}}\right)}{N} \end{array}$$ (6)

where, as in Equations 5, 6, $\theta_{j}*$ is the scaled form-Y ability, and θ_*Y*_*J*__ and θ_*X*_*J*__ are person j's abilities on forms Y and X, and N is the number of common examinees. Second, to substantiate the Rasch analysis, Infit and Outfit mean-square (MNSQ) statistics for every item were obtained from the Winsteps calibration. In addition, the dimensionality of each form was examined in R (R Core Team, 2024) using the psych package (Revelle, 2024): tetrachoric inter-item correlation matrices were computed, and their eigenvalues—the first and second eigenvalues, their ratio, and the proportion of variance explained—were inspected together with a principal-axis factor analysis and Horn's parallel analysis, separately for paper-based and computer-based forms. Third, the agreement between separate and concurrent calibration was evaluated at the item-parameter level (Table 4 and Figure 2). Finally, cross-form test information functions (TIFs) were compared to assess measurement precision across modes. To place the equating on an inferential footing, we computed 95% percentile bootstrap confidence intervals in R by resampling the full common examinees 2,000 times and recomputing A, B, RMSE, and Δ in each resample, and we tested the equivalence of Δ with two one-sided tests (TOST; Lakens, 2017; Efron and Tibshirani, 1993). The TOST null hypotheses are that the true difference is at least +δ or at most –δ; equivalence is concluded when both are rejected. Two margins were used: δ = 0.5 logits (the “Differences That Matter” heuristic) and δ = 0.2 logits, approximately the conditional standard error of measurement near the center of the scale. Because only the four paper–computer pairs estimate a mode difference, the equivalence inference is based on those pairs; the two electronic–electronic pairs serve as same-mode benchmarks.

**Figure 2:**
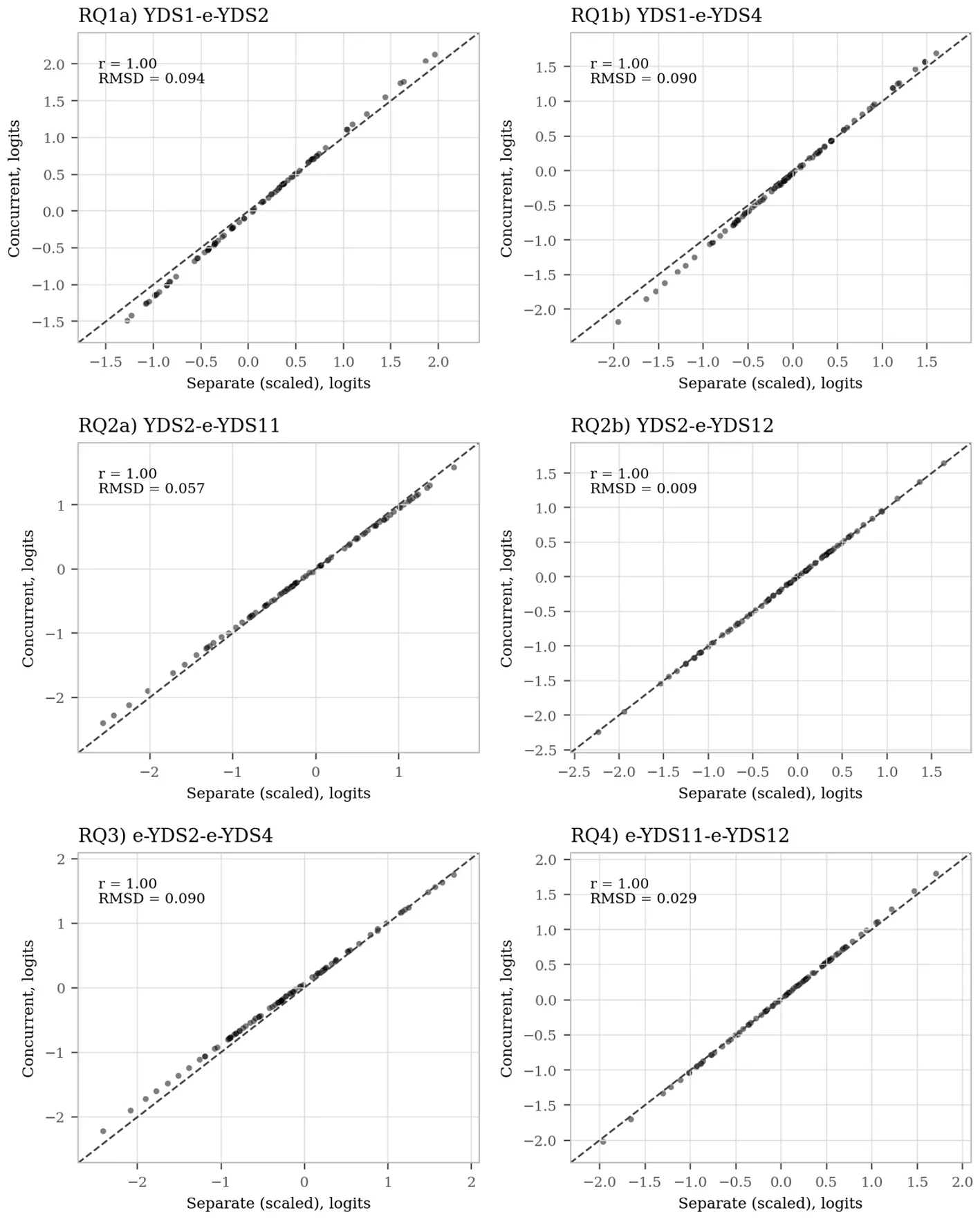
Form-Y item difficulties from separate calibration (scaled to the reference form) vs. concurrent calibration, for each pair. Points lie on the identity line (dashed); *r* = 1.00 in every pair.

**Table 4:** Agreement of form-Y item difficulties between separate (scaled) and concurrent calibration.

Pair	Comparison	*r*	Item-parameter RMSD (logits)
RQ1a	YDS1–e-YDS2	1.00	0.094
RQ1b	YDS1–e-YDS4	1.00	0.090
RQ2a	YDS2–e-YDS11	1.00	0.057
RQ2b	YDS2–e-YDS12	1.00	0.009
RQ3	e-YDS2–e-YDS4	1.00	0.090
RQ4	e-YDS11–e-YDS12	1.00	0.029

## Results

## Model fit and dimensionality

Across all forms, mean Infit and Outfit MNSQ values were close to 1.00, and only a small number of items (0–3 per 80-item form under the 0.5–1.5 productive-range criterion) showed misfit (Table 5). Dimensionality was examined on the tetrachoric inter-item correlations of each form. The first eigenvalue was 8.7–12.6 times larger than the second across all forms (λ1 ≈ 25–30 vs. λ2 ≈ 2.1–3.0), and the first factor accounted for roughly 32–38% of the variance whereas the second accounted for only about 3% (Table 6). This pattern—one large eigenvalue followed by a series of much smaller, similar ones—indicates a dominant single dimension in every form. Horn's parallel analysis retained two factors, because the second eigenvalue slightly exceeded its random reference; however, this secondary factor was negligible relative to the first and is consistent with the minor subsection structure (e.g., grammar, vocabulary, reading) typical of language proficiency tests. Importantly, the dimensional profile was equivalent for the paper-based (YDS) and computer-based (e-YDS) forms—comparable eigenvalue ratios and explained variance—indicating that the computer-based forms did not display a clearly stronger secondary dimension than the paper-based forms. Because the forms also differ in items and administration date, this pattern is consistent with—but does not by itself isolate—invariance of dimensionality across mode; it nonetheless supports the essential unidimensionality required by the Rasch model.

**Table 5:** Item fit (Infit and outfit MNSQ) by form.

Pair	Form	Infit M	Infit range	Outfit M	Outfit range	Misfit (0.5–1.5)	*N*
RQ1a	YDS1	1.00	0.81–1.29	1.01	0.69–1.81	1	4,387
	e-YDS2	1.00	0.81–1.37	1.00	0.56–1.75	3	3,572
RQ1b	YDS1	1.00	0.79–1.29	1.01	0.68–1.73	2	4,699
	e-YDS4	1.00	0.83–1.51	1.02	0.66–2.11	2	3,605
RQ3	e-YDS2	1.00	0.80–1.37	1.00	0.56–1.79	3	3,225
	e-YDS4	1.00	0.83–1.52	1.01	0.66–2.13	2	3,198
RQ2a	YDS2	1.00	0.81–1.40	1.02	0.74–1.78	3	4,817
	e-YDS11	0.99	0.78–1.32	1.00	0.58–1.53	1	3,565
RQ2b	YDS2	1.00	0.82–1.35	1.02	0.75–1.73	2	4,957
	e-YDS12	1.00	0.78–1.31	1.01	0.70–1.44	0	3,840
RQ4	e-YDS11	0.99	0.78–1.32	1.00	0.57–1.53	1	3,209
	e-YDS12	1.00	0.78–1.32	1.01	0.70–1.45	0	3,343

**Table 6:** Dimensionality evidence by form (eigenvalues of the tetrachoric inter-item correlation matrix).

Pair	Form	λ_1_	λ_2_	λ_1_/λ_2_	1st factor var %	2nd factor var %	Parallel analysis
RQ1a	YDS1	26.4	2.14	12.3	33.0	2.7	2
RQ1a	e-YDS2	30.0	2.69	11.2	37.5	3.4	**2**
RQ1b	YDS1	26.4	2.10	12.6	33.0	2.6	**2**
RQ1b	e-YDS4	26.2	2.99	8.8	32.8	3.7	**2**
RQ2a	YDS2	27.9	2.27	12.3	34.9	2.8	**2**
RQ2a	e-YDS11	26.0	2.63	9.9	32.5	3.3	**2**
RQ2b	YDS2	26.4	2.29	11.5	33.0	2.9	**2**
RQ2b	e-YDS12	25.4	2.54	10.0	31.8	3.2	**2**
RQ3	e-YDS2	30.3	2.69	11.3	37.9	3.4	**2**
RQ3	e-YDS4	26.4	3.05	8.7	33.0	3.8	**2**
RQ4	e-YDS11	26.2	2.69	9.7	32.8	3.4	**2**
RQ4	e-YDS12	26.0	2.58	10.1	32.5	3.2	**2**

λ_1_, λ_2_ = first and second eigenvalues of the tetrachoric correlation matrix; variance % = λ/number of items. Parallel analysis used principal-axis extraction with tetrachoric correlations.

## Scaling constants and linking discrepancies

Table 7 reports, for separate calibration on the common examinees, the scaling constants (A, B), the linking RMSE, and the paired mean difference Δ = mean(θ_X_−θ_Y_). The A coefficients ranged from about 0.89 to 1.08, indicating comparable score variability across forms, and the B coefficients ranged from about −0.20 to 0.09. The linking RMSE (the person-level spread between the scaled and the original form-Y estimates, i.e., the magnitude of the change introduced by the transformation) ranged from about 0.04 to 0.17 logits. For the four paper–computer pairs, the paired mean difference Δ ranged from −0.17 to 0.04 logits; the predominantly negative values indicate that the computer-based form yielded, on average, slightly higher ability estimates. The two electronic–electronic pairs (RQ3, RQ4) index between-form and between-session variation under a constant mode. The concurrent solution is not summarized by a separate error metric here; its agreement with separate calibration is instead reported at the item-parameter level (Table 4 and Figure 2).

**Table 7:** Scaling Constants (A, B), linking RMSE, and paired mean difference on the common examinees (separate calibration).

Pair	Form X	Form Y	A	B	RMSE	Paired mean diff. Δ (θ_X_ – θ_Y_)
RQ1a	YDS1	e-YDS2	0.885	0.088	0.125	0.040
RQ1b	YDS1	e-YDS4	0.897	−0.057	0.153	−0.114
RQ2a	YDS2	e-YDS11	1.082	−0.118	0.116	−0.087
RQ2b	YDS2	e-YDS12	0.999	−0.173	0.173	−0.173
RQ3	e-YDS2	e-YDS4	1.062	−0.203	0.169	−0.158
RQ4	e-YDS11	e-YDS12	0.958	0.003	0.044	−0.020

## Sensitivity to exclusion of inconsistent examinees

Because excluding examinees whose scores diverge across modes could, in principle, remove the very mode effect under study, we compared the linking on the full common-person sample (the primary analysis) with that on the trimmed sample. The exclusion changed the results negligibly: the common-person correlations rose by about 0.02 (e.g., from 0.90 to 0.92), whereas the scaling constants A and B and—critically—the estimated mode difference (the mean ability gap between forms) were essentially unchanged (e.g., 0.17 vs. 0.16 logits; Table 3). Because the mode-difference estimate that the study exists to recover does not depend on the exclusion, the primary results are reported on the full sample, with the trimmed results serving as a robustness check.

## Bootstrap confidence intervals and equivalence testing

To place the equating on an inferential footing, we computed 95% bootstrap confidence intervals (2,000 resamples of the full common examinees) for the scaling constants and linking metrics, and tested the equivalence of the paired mean difference Δ against ±0.5- and ±0.2-logit margins with two one-sided tests (TOST; Lakens, 2017). Table 8 reports the intervals and equivalence tests. For the four cross-mode (paper–computer) pairs the intervals were narrow: A ranged from 0.89 to 1.08, and the paired mean difference Δ did not exceed about 0.17 logits in absolute value. Equivalence was supported for all four cross-mode pairs at both the ±0.5-logit margin (TOST *p* < 0.001) and the stricter ±0.2-logit margin (all *p* ≤ 0.001). This tests whether the pre-linking systematic form-plus-mode shift (the location difference the linking corrects) is smaller than a negligible margin, not the post-linking residual, which is zero by construction. Because ±0.2 logits is approximately the conditional standard error of measurement near the center of the scale, differences smaller than this margin fall within measurement error; we nonetheless caution that, for a high-stakes examination, even a sub-threshold difference could matter at a particular classification cut-score, so the equivalence claim applies to the ability continuum as a whole rather than to any specific decision boundary. The two same-mode (electronic–electronic) pairs, RQ3 and RQ4, do not estimate a mode difference and are reported only as benchmarks for between-form and between-session variation under a constant mode.

**Table 8:** Bootstrap 95% confidence intervals and TOST equivalence tests for the paired mean difference (Δ).

Pair	Comparison	Mode	A [95% CI]	B [95% CI]	RMSE [95% CI]	Δ [95% CI]	TOST *p* (±0.5)	TOST *p* (±0.2)
RQ1a	YDS1–e-YDS2	cross	0.885 [0.862, 0.907]	0.088 [0.068, 0.108]	0.125 [0.103, 0.148]	0.040 [0.019, 0.061]	< 0.001	< 0.001
RQ1b	YDS1–e-YDS4	cross	0.897 [0.879, 0.915]	−0.057 [−0.075, −0.039]	0.153 [0.133, 0.172]	−0.114 [−0.132, −0.096]	< 0.001	< 0.001
RQ2a	YDS2–e-YDS11	cross	1.082 [1.059, 1.104]	−0.118 [−0.139, −0.098]	0.116 [0.098, 0.134]	−0.087 [-0.106, −0.068]	< 0.001	< 0.001
RQ2b	YDS2–e-YDS12	cross	0.999 [0.979, 1.019]	−0.173 [−0.191, −0.155]	0.173 [0.157, 0.190]	−0.173 [−0.190, −0.157]	< 0.001	0.001
RQ3	e-YDS2–e-YDS4	same	1.062 [1.024, 1.104]	−0.203 [−0.243, −0.165]	0.169 [0.140, 0.200]	−0.158 [−0.190, −0.125]	< 0.001	0.006
RQ4	e-YDS11–e-YDS12	same	0.958 [0.925, 0.992]	0.003 [−0.026, 0.030]	0.044 [0.014, 0.080]	−0.020 [−0.048, 0.007]	< 0.001	< 0.001

“Mode”, cross (paper vs. computer) or same (electronic vs. electronic). Only the four cross-mode pairs estimate a mode difference; RQ3 and RQ4 are same-mode benchmarks. Δ, paired mean difference (mean θ_*X*_ – mean θ_*Y*_) on the common examinees. Margins: ±0.5 logits (DTM heuristic) and ±0.2 logits (≈ conditional SEM) Δ is the pre-linking systematic between-form (form-plus-mode) difference—the location shift the linking corrects—not a post-linking residual (which is zero by construction); the TOST tests whether this systematic shift falls within the margin.

## Agreement between separate and concurrent calibration

**Figure 3:**
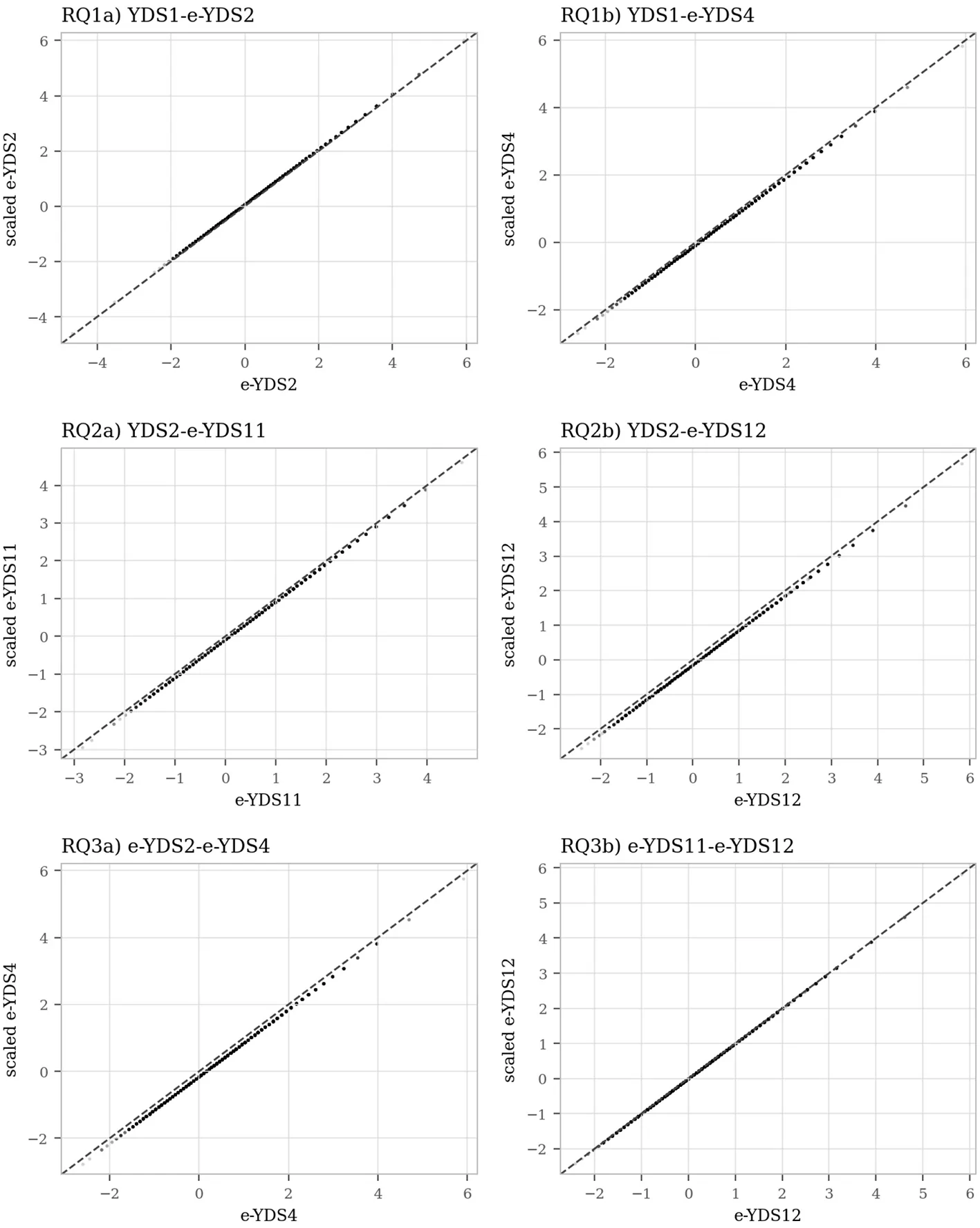
Concurrently estimated vs. separately estimated ability parameters (six pairs).

**Figure 4:**
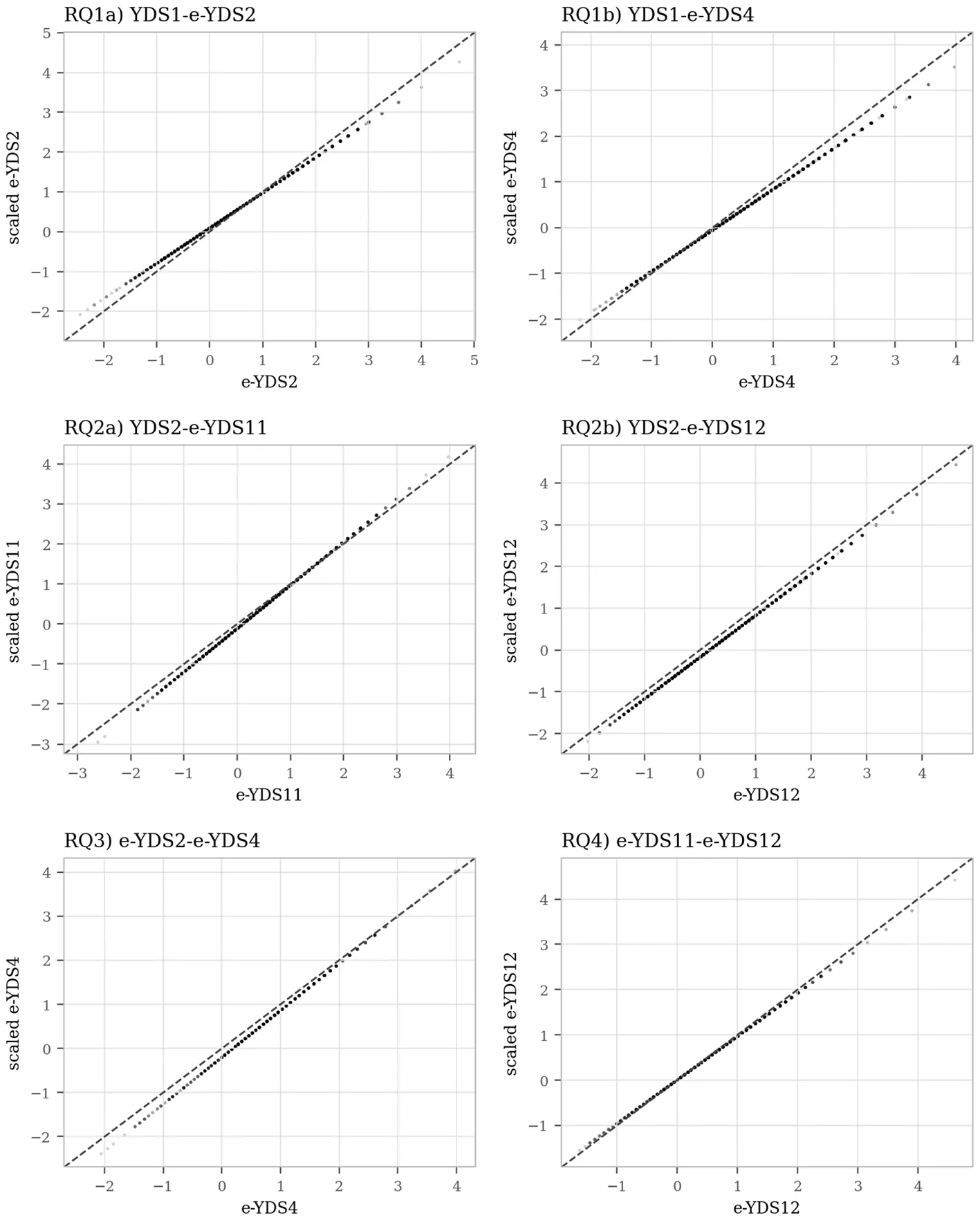
Estimated vs. scaled ability parameters under separate calibration (six pairs).

To make the item-parameter agreement explicit, the concurrently estimated form-Y item difficulties were expressed on the reference (form-X) scale and compared with the separately estimated, transformed difficulties. The two solutions agreed almost perfectly for every pair (*r* = 1.00; item-parameter RMSD = 0.009–0.094 logits; Table 4 and Figure 2), indicating that the common scale is recovered consistently regardless of estimation route. This item-level comparison is distinct from Figure 3, which displays the agreement of person ability estimates under concurrent calibration; the two analyses concern different parameters (items vs. persons) and should not be conflated. The scatter of estimated against scaled abilities fell closely along the identity line for every pair (Figure 4, separate calibration; Figure 3, concurrent calibration), with the e-YDS11–e-YDS12 pair showing the tightest alignment. In Figure 3, because concurrent calibration involves no post-hoc scaling, the panels show agreement of the two ability estimates rather than a scaling transformation.

## Cross-form test information

**Figure 5:**
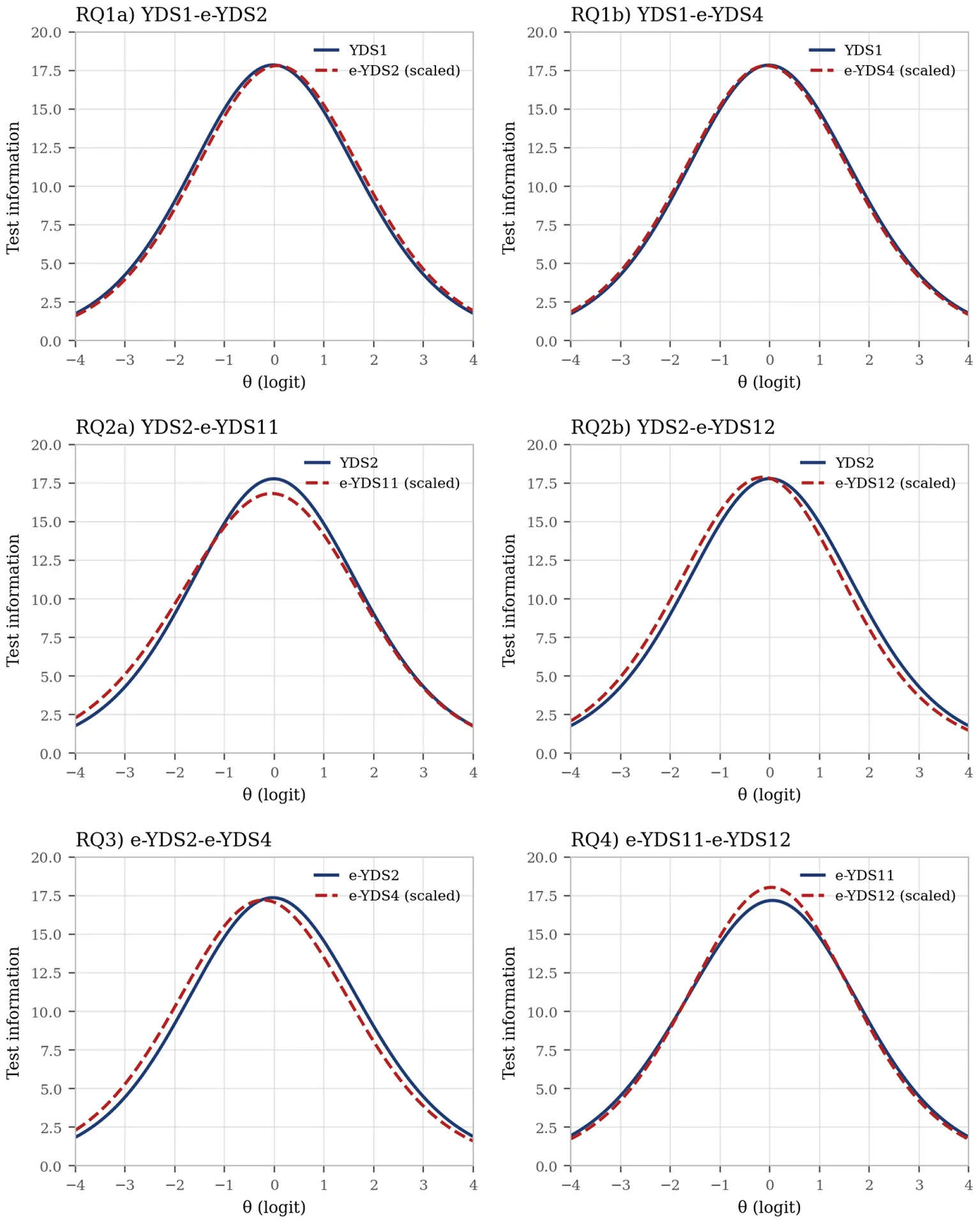
Cross-form test information functions under separate calibration: each paper-based or reference form vs. the linked computer-based form expressed on the common scale (dashed).

**Figure 6:**
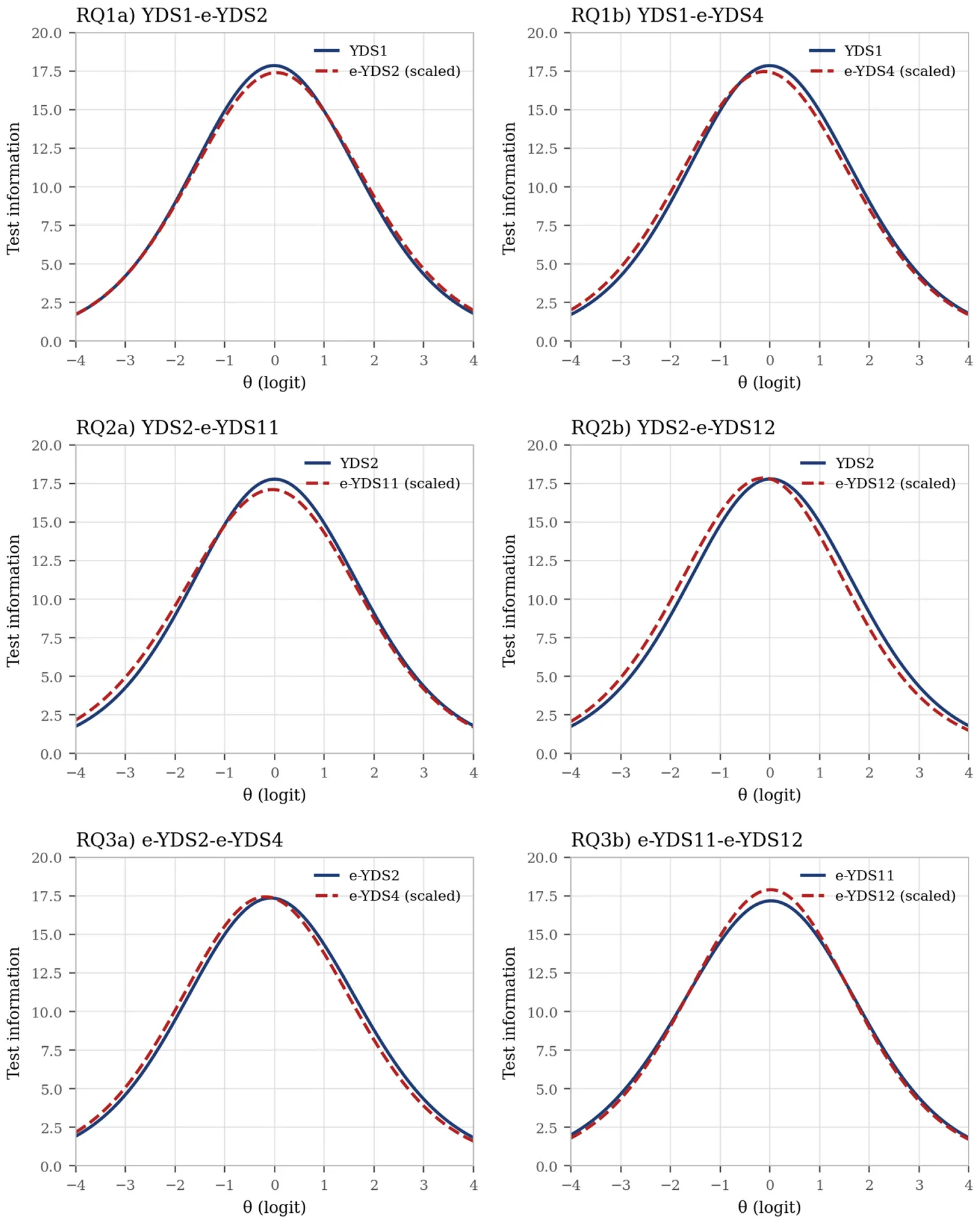
Cross-form test information functions under concurrent calibration, with both forms on the single concurrent scale.

Test information functions were used to compare measurement precision across modes. We note that information functions computed from a single form's item parameters will force the “observed” and “scaled” curves to coincide, because—absent common items—both would be derived from the same item set; such overlap is an artifact of comparing a form with itself rather than evidence of cross-form equivalence. We therefore computed genuine cross-form TIFs, contrasting form-X item difficulties with form-Y item difficulties expressed on the common scale. Under separate calibration (Figure 5) the two forms provided similar but not identical information: peak information differed by at most about one information unit (peaks ≈ 17–18) and the maximum absol ute difference across the ability range was 0.4–1.2 information units, depending on the pair. The concurrently calibrated item parameters, which lie on a single scale by construction, yielded the same picture (Figure 6). We interpret these functions as evidence of comparable measurement precision across modes, while noting that similarity of information is distinct from, and does not by itself establish, score equating.

## Discussion

This study placed six paper-based and computer-based form pairs of a high-stakes language examination on a common scale under a common-person design, in order to assess mode comparability rather than to perform a routine form equating. Across pairs, the data fit the Rasch model adequately and were essentially unidimensional, with a comparably dominant single dimension for paper-based and computer-based forms; linking constants were stable; the estimated mode differences were small ( ≤ ~0.17 logits); and the separate and concurrent methods recovered the same common scale (*r* = 1.00).

Equating results are known to depend on sample size (Livingston and Kim, 2010), group ability differences (Chen et al., 2010), item-difficulty differences across forms (Ricker and von Davier, 2007; Chu, 2002), and the proportion of common elements (Tong and Kolen, 2005). Consistent with this, lower error metrics were observed where the proportion of common examinees was higher and ability differences between forms smaller. The separate and concurrent calibrations recovered the same common scale, agreeing almost perfectly at the item-parameter level (*r* = 1.00; Table 4), consistent with prior research on concurrent estimation (Hanson and Béguin, 2002; Kim, 2019).

Computer-based forms tended to yield slightly higher ability estimates, reflected in the predominantly negative B constants; the common-examinee distributions nonetheless remained stable. We are cautious about characterizing these differences purely in logits. On an 80-item test, a shift of 0.5 logits corresponds to roughly 8–9 number-correct points near the center of the score scale—where test information, and hence the slope of the test characteristic curve, is about 17–18—and would be far from negligible at a classification cutoff. We also note that the 0.5-logit “Differences That Matter” threshold (Dorans and Feigenbaum, 1994) was proposed in the SAT context and does not necessarily transfer to an 80-item, 180-min language test; we therefore complement it with the formal equivalence tests reported above (Table 8). The relevant point is therefore not that this threshold is small, but that the discrepancies actually observed here are an order of magnitude smaller: the paired mean difference did not exceed about 0.17 logits, corresponding to at most roughly 3 number-correct points near the center and fewer toward the extremes. We thus regard the observed differences as practically minor for the typical examinee, while acknowledging that even a few points can matter for examinees near a decision boundary.

The similarity of cross-form test information indicates comparable measurement precision across modes, but we treat precision and score comparability as distinct properties and do not infer the latter from the former. The small mode differences may reflect both effective standardization of the computer-based environment and genuine residual mode effects—interface, scrolling, or screen-fatigue differences—that cannot be entirely discounted (Fishbein et al., 2018; Jerrim et al., 2018). The slightly higher computer-based estimates may also stem partly from the more selective e-YDS population.

Our findings are most accurately described as evidence of mode comparability rather than as a demonstration of a required equating. They are consistent with the position that, when a mode-comparability study finds no substantial mode effect, a testing program may treat the two modes as comparable without imposing a *post-hoc* score linking (Pommerich, 2016). We emphasize, however, that the present design does not fully separate mode effects from form, item, temporal, and selection effects, so this evidence supports practical comparability under the conditions studied rather than a pure mode equivalence. The contribution of this study is to provide the empirical basis for that decision; whether ÖSYM ultimately links scores or treats them as already comparable is a policy choice that the present evidence supports but does not, by itself, dictate. Continued monitoring remains advisable as digital delivery evolves.

More broadly, the study illustrates how rigorous design and standardized administration support replicable, trustworthy inference in high-stakes testing. By verifying examinee identity and standardizing conditions, it reduces sources of variability that often threaten reproducibility, underscoring that systematic design and careful procedure are central to the credibility of assessment results.

## Limitations

Several limitations qualify these conclusions. First, because the paper- and computer-based forms share no common items, any observed difference reflects a confound of mode with form- and item-difficulty differences; the common-person design isolates mode only under the assumption that the same examinees anchor the scales, and a pure mode effect cannot be fully separated from form-specific item differences. Second, the linked administrations were 15–76 days apart, so genuine change in examinee ability—through study, practice, or fatigue—may contribute to the observed differences. Third, the e-YDS is offered only in a limited number of urban centers and may attract a somewhat more prepared or motivated population, so selection rather than mode alone may partly explain the slightly higher electronic estimates. Fourth, demographic and motivational variables were not matched across forms, and interface features (layout, scrolling, font size) were not manipulated experimentally. Fifth, although the direction of administration (paper-to-electronic vs. electronic-to-paper) was built into the design, order effects were not formally tested and the B constants were not modeled as a function of administration order; this should be regarded as a limitation rather than a controlled design feature. Sixth, because the forms share no common items, item-level differential item functioning (DIF) across modes could not be evaluated, so strict measurement invariance was not established. Seventh, the data come from 2024 administrations only; possible drift in form characteristics over time, and the self-selection of e-YDS examinees, were not modeled. Finally, the analysis was limited to the English version of the YDS, so generalization to other languages, populations, or interactive digital formats should be made with caution.

## Future Research

Future work should match examinees on demographic and background variables, extend the design to other language versions, and manipulate interface features experimentally to isolate their effects. Replication across test types and populations would further strengthen generalizability.

## Data Availability

The data that support the findings of this study are subject to restrictions imposed by third-party ownership and are not publicly available. Inquiries regarding the dataset can be directed to tubagunduz@mu.edu.tr.

## References

[B1] American Educational Research Association (2014) “American Psychological Association and National Council on Measurement in Education,” Standards for Educational and Psychological Testing (Washington DC: AERA)..

[B2] Bennett R. E. (2003). Online Assessment and the Comparability of Score Meaning. Princeton, NJ: Educational Testing Service.

[B3] Boone W. J. Staver J. R. (2020). “Common person test equating,” in Advances in Rasch Analyses in the Human Sciences (Springer), 147–158. doi: 10.1007/978-3-030-43420-5_11

[B4] Breiter A. Groß L. M. Stauke E. (2013). “Computer-based large-scale assessments in Germany,” in Next Generation of Information Technology in Educational Management, ed. D. Passey (Heidelberg: Springer), (IFIP AICT, vol. 400), 41–54. doi: 10.1007/978-3-642-38411-0_4

[B5] Chen H. Cui Z. Zhu R. Gao X. (2010). Evaluating the Effects of Differences in Group Abilities on the Tucker and the Levine Observed-Score Methods for Common-Item Nonequivalent Groups Equating (ACT Research Report 2010-1). City, IA: ACT. doi: 10.1037/e548302011-001

[B6] Chu K. L. (2002). Equivalent Group Test Equating with the Presence of Differential Item Functioning (Doctoral dissertation). Tallahassee, FL: Florida State University.

[B7] Cook L. L. Eignor D. R. (1991). IRT equating methods. Educ. Meas.: Issues Pract. 10, 37–45. doi: 10.1111/j.1745-3992.1991.tb00207.x

[B8] Dorans N. J. (2004). Equating, concordance, and expectation. Appl. Psychol. Meas. 28, 227–46. doi: 10.1177/0146621604265031

[B9] Dorans N. J. Feigenbaum M. D. (1994). “Equating issues engendered by changes to the SAT and PSAT/NMSQT,” in Technical Issues Related to the Introduction of the New SAT and PSAT/NMSQT, eds. I. M. Lawrence, N. J. Dorans, M. D. Feigenbaum, N. J. Feryok, A. P. Schmitt, and N. K. Wright (Princeton, NJ: Educational Testing Service), (ETS Research Memorandum No. RM-94-10), 91–122.

[B10] Efron B. Tibshirani R. J. (1993). An Introduction to the Bootstrap. New York, NY: Chapman and Hall/CRC. doi: 10.1007/978-1-4899-4541-9

[B11] Fishbein B. Martin M. O. Mullis I. V. S. Foy P. (2018). The TIMSS 2019 item equivalence study. Large Scale Assess. Educ. 6, 1–23. doi: 10.1186/s40536-018-0064-z

[B12] Hanson B. A. Béguin A. A. (1999). Separate Vs. Concurrent Estimation of IRT Item Parameters in the Common Item Equating Design (ACT Research Report). Iowa City, IA: ACT. doi: 10.1037/e427762008-001

[B13] Hanson B. A. Béguin A. A. (2002). Obtaining a common scale for IRT item parameters using separate vs. concurrent estimation in the common-item equating design. Appl. Psychol. Meas. 26, 3–24. doi: 10.1177/0146621602026001001

[B14] Holland P. W. Dorans N. J. (2006). “Linking and equating,” in Educational Measurement, 4th Edn., ed. R. L. Brennan (Westport, CT: Praeger), 187–220.

[B15] Jerrim J. Micklewright J. Heine J. Salzer C. McKeown C. (2018). PISA 2015: How big is the “mode effect” and what has been done about it? Oxf. Rev. Educ. 44, 476–93. doi: 10.1080/03054985.2018.1430025

[B16] Karkenova A. (2025). Advancing digital item development in Asian assessment systems: insights from Kazakhstan's Unified National Testing. Front. Educ. 10:1654674. doi: 10.3389/feduc.2025.1654674

[B17] Kim K. Y. (2019). A comparison of the separate and concurrent calibration methods for the full-information bifactor model. Appl. Psychol. Meas. 43, 512–26. doi: 10.1177/0146621618813095 PMC6739745 31534287

[B18] Kingston N. M. (2009). Comparability of computer- and paper-administered multiple-choice tests for K−12 populations: a synthesis. Appl. Meas. Educ. 22, 22–37. doi: 10.1080/08957340802558326

[B19] Kolen M. J. Brennan R. L. (2004). Test Equating, Scaling, and Linking: methods and Practices, 2nd edn. New York, NY: Springer. doi: 10.1007/978-1-4757-4310-4

[B20] Kolen M. J. Brennan R. L. (2014). Test Equating, Scaling, and Linking: methods and Practices, 3rd edn. New York, NY: Springer. doi: 10.1007/978-1-4939-0317-7

[B21] Koomen M. Zoanetti N. (2016). Strategic planning tools for large-scale technology-based assessments. Assess. Educ. 25, 200–23. doi: 10.1080/0969594X.2016.1173013

[B22] Lakens D. (2017). Equivalence tests: a practical primer for t tests, correlations, and meta-analyses. Soc. Psychol. Personal. Sci. 8, 355–62. doi: 10.1177/1948550617697177 PMC5502906 28736600

[B23] Linacre J. M. (2011a). A user's Guide to WINSTEPS/MINISTEPS [Computer Software]. Beaverton, OR: Winsteps.com.

[B24] Linacre J. M. (2011b). Winsteps Rasch Measurement and Rasch Analysis Software [Computer software]. Available online at: http://www.winsteps.com (Accessed August 28, 2026).

[B25] Liu J. Jiang Z. Zheng T. Han Y. Feng S. (2025). Common persons design in score equating: a Monte Carlo investigation. Educ. Psychol. Meas. 86, 320–44. doi: 10.1177/00131644251380585 PMC12571793 41180323

[B26] Livingston S. A. Kim S. (2010). Random-groups equating with samples of 50 to 400 test takers. J. Educ. Measure. 47, 175–85. doi: 10.1111/j.1745-3984.2010.00107.x

[B27] Ma S. Krötz M. Deutscher V. Winther E. (2025). Same same yet different: equivalence of the multidimensional assessment of vocational competence across test formats. Int. J. Train Dev. 29, 433–48. doi: 10.1111/ijtd.12372

[B28] ÖSYM (2025a). Foreign Language Examination (YDS/1) Guide. Center for Assessment, Selection and Placement. Available online at: https://dokuman.osym.gov.tr/pdfdokuman/2025/YDS-INGILIZCE/kilavuz_ydingd20052025.pdf (Accessed August 28, 2026).

[B29] ÖSYM (2025b). Electronic Foreign Language Examination (e-YDS) Guide. Center for Assessment, Selection and Placement. Available online at: https://dokuman.osym.gov.tr/pdfdokuman/2025/e-YDS/kilavuz_e-ydd15042025.pdf (Accessed August 28, 2026).

[B30] Pai H. (2025). Presidential address 2025: expansion of computer-based testing from 12 to 27 health professions by 2027 and adoption of a large language model for item generation. J. Educ. Eval. Health Prof. 22:7. doi: 10.3352/jeehp.2025.22.7 PMC11934035 39833983

[B31] Perry K. Meissel K. Hill M. F. (2022). Rebooting assessment: Exploring the challenges and benefits of shifting from pen-and-paper to computer in summative assessment. Educ. Res. Rev. 36:100451. doi: 10.1016/j.edurev.2022.100451

[B32] Pommerich M. (2016). “The fairness of comparing test scores across different tests or modes of administration,” in Linking and Aligning Scores and Scales, eds. N. J. Dorans and L. L. Cook (Routledge), 111–34. doi,: 10.4324/9781315774527-9

[B33] R Core Team (2024). R: A Language and Environment for Statistical Computing [Computer software]. Vienna: R Foundation for Statistical Computing. Available online at: https://www.R-project.org/ (Accessed August 28, 2026).

[B34] Revelle W. (2024). Psych: procedures for Psychological, Psychometric, and Personality Research (R package version 2.4). Evanston, IL: Northwestern University. Available online at: https://CRAN.R-project.org/package=psych (Accessed August 28, 2026).

[B35] Ricker K. L. von Davier A. A. (2007). The impact of anchor test length on equating results in a nonequivalent groups design. ETS Res. Rep. Series 2007, i−19. doi: 10.1002/j.2333-8504.2007.tb02086.x

[B36] Tong Y. Kolen M. J. (2005). Assessing equating results on different equating criteria. Appl. Psychol. Meas. 29, 418–32. doi: 10.1177/0146621606280071

[B37] Tuna Pusa E. Dinçer S. (2025). A meta-synthesis study on the use of e-assessment tools in education. SAGE Open 15, 1–18. doi: 10.1177/21582440251360495

[B38] van der Linden W. J. Hambleton R. K. (1997). Handbook of Modern Item Response Theory. New York, NY: Springer. doi: 10.1007/978-1-4757-2691-6

[B39] von Davier M. Foy P. Martin M. O. Mullis I. V. S. (2020). “Examining eTIMSS country differences between eTIMSS data and bridge data,” in TIMSS 2019 Technical Report, eds. M. O. Martin, M. von Davier, and I. V. S. Mullis (Chestnut Hill, MA: TIMSS and PIRLS International Study Center, Boston College), 13.1–13.24.

[B40] Wang S. Jiao H. Young M. J. Brooks T. Olson J. (2007). Comparability of computer-based and paper-and-pencil testing in K−12 reading assessments. Educ. Psychol. Meas. 68, 5–24. doi: 10.1177/0013164407305592

[B41] Wei S. Liu X. Jia Y. (2014). Using Rasch measurement to validate the instrument of students' understanding of models in science (SUMS). Int. J. Sci. Math. Educ. 12, 1067–82. doi: 10.1007/s10763-013-9459-z

[B42] Wiberg M. Laukaityte I. (2025). Calculating bias in test score equating in a NEAT design. Appl. Psychol. Meas. 49, 350–66. doi: 10.1177/01466216251330305 PMC11948241 40162326

[B43] Wu T. Kim S. Y. Westine C. Boyer M. (2025). IRT observed-score equating for rater-mediated assessments using a hierarchical rater model. J. Educ. Meas. 62, 145–71. doi: 10.1111/jedm.12425

[B44] Yu C. H. Popp S. O. (2005). Test equating by common items and common subjects: concepts and applications. Pract. Assess. Res. Eval. 10:4. doi: 10.7275/68dy-z131

[B45] Zhai X. Pellegrino J. W. (2023). “Large-scale assessment in science education,” in Handbook of Research on Science Education, eds. N. G. Lederman, D.L. Zeidler, and J.S. Lederman (New York, NY: Routledge) (Volume III), 1045–97. doi: 10.4324/9780367855758-38

[B46] Zhu W. (1998). Test equating: what, why, how? Res. Q. Exerc. Sport. 69, 11–23. doi: 10.1080/02701367.1998.10607662 9532618

